# Implementation of a peer support intervention for family members of involuntarily hospitalised patients

**DOI:** 10.1192/j.eurpsy.2024.1244

**Published:** 2024-08-27

**Authors:** I. Wells, K. Wintsch, D. Giacco

**Affiliations:** ^1^University of Warwick, Coventry; ^2^Queen Mary University of London, London, United Kingdom

## Abstract

**Introduction:**

Peer support has been identified as successful in improving patient wellbeing and empowerment and there is evidence that peer support can also help their family members (FMs). A peer support programme for FMs, developed in Germany, significantly improved FMs’ quality of life. We have worked to adapt this support programme for delivery in England. We will report the results of this adaptation process and of the implementation of peer support for FMs.

**Objectives:**

To examine the feasibility of the peer support programme developed and assess whether it can be delivered using a “train-the-trainer” approach.

**Methods:**

The peer support programme is being implemented in two stages. In stage one, FMs with experience of supporting an involuntarily hospitalised patient (family peer supporters (FPSs)) receive an online training programme consisting of four sessions. These sessions, provided by the research team and FMs with lived experience of caring for an involuntarily hospitalised patient, teach FPSs skills in communication, reflection, coping and trialogue (promotion of equal communication between FMs, professionals and patients). FPSs then use these skills to deliver support to FMs of patients who are currently involuntarily hospitalised. This support is delivered for up to three months. The impact of this programme is assessed through one-to-one interviews with FPSs and FMs. Questionnaires are also provided to FMs measuring their quality of life, caregiving burden and psychological wellbeing before and after receiving support from FPSs.

In stage two, a modified version of the training programme (based on FPS feedback) is provided to a new group of FPSs. This training will be delivered by FPSs from stage one. After receiving the training programme, stage two FPSs will deliver support to other FMs as described for stage one. Assessment of the modified programme will mirror stage one.

**Results:**

Provision of the stage one training programme is complete, and delivery of support is in progress with modifications being made for stage two. Eight FPSs and six out of a target of 12 FMs have been recruited for stage one. FPSs reported the training programme to be a positive experience, highlighting that it was engaging, easy to understand and gave them the confidence to support other FMs. Technical difficulties and an overload of information were cited as areas to improve for the next stage.

**Conclusions:**

FPSs described the peer support training programme as a positive experience overall. However, improvements need to be made for stage two which is in progress. A more comprehensive report of our findings, including the impact of this peer support programme and the feasibility of implementing it in England, will be provided as the programme progresses.

**Disclosure of Interest:**

None Declared

